# Biological Complexity and Cryptographic Secrecy: Reproducible Key Recovery and a Structured Evidence Audit of Bio-Derived Key Schemes

**DOI:** 10.3390/biomimetics11100741

**Published:** 2026-10-15

**Authors:** Abdullah Alabdulatif

**Affiliations:** Department of Computer Science, College of Computer, Qassim University, Buraydah 52571, Saudi Arabia; a.alabdulatif@qu.edu.sa

**Keywords:** bio-inspired cryptography, DNA cryptography, protein-based key derivation, fuzzy extractors, min-entropy, key recovery, structured evidence audit, reproducibility

## Abstract

Bio-derived key schemes build cryptographic keys from biological material: protein structures, DNA and RNA sequences, amino-acid chains, proteinoid assemblies. This paper audits the class against a protocol drafted prospectively, refined during a pre-specified pilot, and frozen before the main screening and extraction, and tests its central claim empirically. No audited study reported a defensible attacker-conditioned min-entropy for its complete secret; the key-space figures instead measured an output or encoding-table size, a descriptor length, or a parameter-range product. A structured evidence audit of twelve studies finds that the biological material rarely holds the secret, that the evidence concerns the randomness of outputs rather than the secrecy of keys, and that no study provided a publicly linked runnable implementation. On three schemes (the two whose secret is a selection from a public set and one encoding scheme) we recover keys from the first two and bound the entropy of the third. The executed recoveries covered a 647-protein candidate set (work factor about 9.3 search-space bits) and a single-reference DNA position search (about 12 bits); a broader eight-reference analysis gives a projected search space upper bound of at most about 39 bits once a 16-bit auxiliary key is included, and was not exhaustively executed. Reported key spaces range up to millions of bits, and for the encoding scheme the input entropy is left unestablished. These results apply to schemes whose secret is public, a selection from public data, or an encoding; they do not extend to a scheme built on a genuinely private biological sample, a noisy physical measurement, or a properly constructed fuzzy extractor. We contribute a taxonomy of biological sources by role, three security observations, the audit and the recovery and accounting experiments, and a design checklist. Biological complexity does not by itself imply cryptographic secrecy.

## 1. Introduction and Research Questions

A growing body of work proposes to build cryptographic keys from biological material: the three-dimensional structure of a protein, a DNA or RNA sequence, an amino-acid chain, or a proteinoid assembly. The appeal is intuitive. Biological objects are intricate, high-dimensional, and often described with large numerical descriptors, and it is tempting to read that intricacy as cryptographic strength. This paper examines that inference directly. Intricacy of a source and secrecy of a key are different properties, and the first does not establish the second.

The distinction matters because a key is only as strong as what an attacker cannot reproduce. If the biological object, or the small set of choices used to pick it, is drawn from a public database, then an attacker with the same public information faces no uncertainty about it, however complex the object is. Security then has to come from somewhere else—a private sample, a random seed, or a noisy measurement the attacker cannot repeat—or it is not present at all. Whether a given scheme meets this requirement is a question that can be answered by reading the scheme against a fixed set of criteria, which is what this study does.

We assess the field on its own terms. We do not assume that bio-derived schemes overclaim, nor that they are sound; we measure the extent to which their security claims are backed by explicit entropy accounting, a defined attacker, and reproducible evidence. Two mature areas provide the reference points a designer would need: the fuzzy-extractor line of work, which separates a noisy source, its min-entropy, public helper data, and a near-uniform extracted key [1,2], and the guidance on entropy sources for random-bit generation, which clearly states that deterministic post-processing cannot manufacture entropy that a source does not contain [3]. These tools are standard in biometric key generation and in physical randomness extraction; the bio-inspired cryptography literature largely does not apply them, and part of this paper is a measurement of that gap. Existing reviews of DNA and bio-inspired cryptography catalogue the designs and their applications [4,5]; they survey schemes on their own terms, whereas a protocol-driven audit that measures the security claims against a defined attacker, with reproducible key-recovery, has been lacking.

This work follows an earlier key-exchange construction by the author that bound protein structure to elliptic-curve Diffie–Hellman [6]. Here we do not propose a construction: we audit the class of schemes that derive keys from biological sources and test the central claim empirically by recovering keys from schemes whose secret is a public structure or a selection from a public set. The paper is an assessment and an attack study, not a new protocol.

Research questions

- RQ1. Which categories of biological source are used for key derivation, and how are published works distributed across them?
- RQ2. To what extent are the actual secret and its guessing space stated explicitly?
- RQ3. To what extent are a defined attacker model and explicit entropy accounting provided?
- RQ4. What proportion of works are reproducible from their published description or from available code?
- RQ5. Do these evidentiary profiles differ by the type of biological source?

Contributions

- C1—a five-category taxonomy of biological sources by the role each can play in key derivation, with the guarantee each role can and cannot provide (Section 4.1).
- C2—three security observations that bound what a biological input can contribute to secrecy (Section 4.2).
- C3—a structured, reproducible evidence audit of the field against six fixed coding axes, with a frozen protocol and reported coding-stability (test–retest) agreement (Section 3, results in Section 5).
- C4—reproducible experiments on three published schemes: key recovery where the secret is a public structure or a public-set selection (measuring success rate, guess count, time, and memory), and an entropy upper-bound for an encoding scheme (Section 6).
- C5—a design checklist that a scheme author can apply before submission (Section 8). The reproducible key-recovery experiments (C4) are the paper’s primary empirical result; the structured audit (C3) frames and motivates them.

Although the methods are cryptographic, the question is central to biomimetics: bio-inspired and bio-conditioned designs routinely borrow the complexity of biological structures and sequences as a security primitive, and this paper tests when that borrowing yields genuine secrecy and when it yields only the appearance of it. The taxonomy and the checklist give designers working in the biomimetic tradition a way to tell the two apart before publication.

## 2. Threat Model and Cryptographic Preliminaries

We fix the terms used throughout and the attacker against whom claims are judged. The setting is a scheme that produces key material K from a biological source and possibly from auxiliary inputs. The question in every case is how much of K an attacker cannot predict.

### 2.1. Attacker Model

Following Kerckhoffs’s principle, we assume the attacker knows the algorithm in full and everything that is public: the design, any fixed tables or encodings, and every biological object or database that the scheme draws on from a public source. The attacker does not know values the scheme keeps secret—a private sample, a random seed, or a per-session nonce. Where a scheme provides a public verification target, such as a ciphertext, a message authentication tag, or a protocol transcript, the attacker is assumed to hold it and to be able to test a candidate key against it. This is the standard model under which key-derivation claims are evaluated, and it is the model a scheme must state if its security is to be assessed.

### 2.2. Secret, Guessing Space, and Min-Entropy

The security-relevant quantity is not the size or length of the biological descriptor but the uncertainty the attacker has about the secret. For a secret S with distribution seen by the attacker, the relevant measure is the min-entropy H∞(S) = −log2 max_s Pr[S = s], and the best single guess succeeds with probability 2^−H∞(S)^. When the secret is a selection of one object from a set of N equally likely public candidates, the ceiling is log2 N bits regardless of how large each object is; a selection from 519 public structures, for instance, provides at most log2(519) ≈ 9.02 bits, with roughly 260 guesses on average [7]. In general H∞(S) ≤ log2|supp(S)|, with equality only under a uniform distribution, where the selection distribution is unknown, log2 N is therefore an upper bound on the secret’s min-entropy—a search-space size, not a measured entropy. We keep this quantity, the attacker’s uncertainty about the secret, strictly separate from the descriptor length and from the size of any public archive the object was sampled from. This (N + 1)/2 figure is a uniform-selection benchmark; an attacker who knew the selection distribution would order candidates by probability and could do better, so it is an upper bound on the optimal expected guesses, not a security guarantee.

### 2.3. Key Derivation, Salt, and Extraction

A key-derivation function maps input keying material to output keys. In the extract-then-expand form [8], an extractor first condenses the entropy of the input into a near-uniform value and an expander then stretches it to the required length; a public salt improves the extractor and separates domains but contributes no secrecy. Fuzzy extractors extend this to noisy sources: they publish helper data P and produce a key, and their analyses account explicitly for how much min-entropy remains in the source given P [1,2]. The relevant lesson for the schemes audited here is structural. A public input, whether a salt, a helper string, or a published biological descriptor, may condition or format a derivation, but it cannot be the source of the secret; the secret must be an input the attacker does not hold.

### 2.4. What Deterministic Processing Cannot Do

Hash functions, encodings, and other deterministic maps redistribute entropy; they do not create it. If the input to such a map is fully known to the attacker, so is the output. A 256-bit hash of a public structure is a public 256-bit value, not a 256-bit secret. This is the operating assumption behind the guidance on entropy sources [3], and it is the single most frequent point of divergence between the schemes audited here and the standard model.

## 3. Structured Audit Protocol

The protocol below was drafted prospectively and refined during a pre-specified pilot that fixed the codebook; it was then frozen before the main screening and extraction, and the pilot studies were re-coded under the frozen codebook. The audit logs and coding materials accompany the paper as Supplementary Material. The pilot served codebook calibration only; every record in the main audited set was coded under the frozen protocol, so no result depends on pre-freeze coding.

### 3.1. Objective

The audit quantifies the extent to which security claims in bio-derived key schemes are supported by explicit entropy accounting, defined attacker models, and reproducible evidence. It measures the extent of support and assumes neither overclaiming nor its absence.

### 3.2. Eligibility

Included: works that propose or implement a scheme in which cryptographic key material is derived from, or selected using, a biological source—sequence, structure, or physical biosignal—including works where the biological component is an encoding or metaphor with no entropy source, since identifying those is part of the study. Excluded: works with no cryptographic or keying claim, such as DNA data storage or steganography without key derivation; bio-inspired metaheuristics used only for parameter tuning; reviews and surveys, which are logged separately for reference chasing and excluded from the denominator; duplicates; and works whose full text is not available for assessment. Biometrics, physical unclonable functions, and physical randomness extraction are read as a reference class only and are excluded from the percentage denominators.

### 3.3. Stratification and Reporting

The DNA-cryptography cluster is large; including it wholesale would make a headline percentage a statement about DNA cryptography under a broader title. The design therefore uses a pre-declared stratification: Stratum A (protein, peptide, amino acid, 3-D structure), retrieved in full by the predefined searches; Stratum B (proteinoid and physical biosignals), retrieved in full; Stratum C (DNA and RNA, published 2015–2026 and indexed in Scopus or Web of Science), retrieved in full where the eligible count does not exceed fifty, and otherwise sampled systematically over ascending publication date with the step and sampling fraction declared. Every constraint is mechanical and set before screening. Results are reported per stratum; a single figure across strata is quoted only when weighted by the sampling fractions, with the weighting stated.

### 3.4. Sources and Search

Six databases were searched (Scopus, IEEE Xplore, Web of Science, Wiley Online Library, DOAJ, and the ACM Digital Library), with arXiv, IACR ePrint, and DBLP as open supporting sources. For each search we record who ran it, the date, the verbatim query, and the result count, and we keep the raw export unchanged. One query family per stratum was used. The full log accompanies the paper. The searches (8–9 September 2026) returned 246 records (Scopus 24, IEEE Xplore 37, Web of Science 100, Wiley 4, DOAJ 56, ACM 25), reduced to 235 after deduplication. A representative Scopus query was TITLE-ABS-KEY((“protein cryptography” OR protein* OR peptide* OR “amino acid” OR AlphaFold) AND (“key generation” OR “key derivation” OR “secret key” OR “key exchange” OR cryptograph* OR encrypt*)), limited to article or conference paper; the full query set, dates, and raw counts are in the search log.

### 3.5. Screening and Extraction

**Figure 1:**
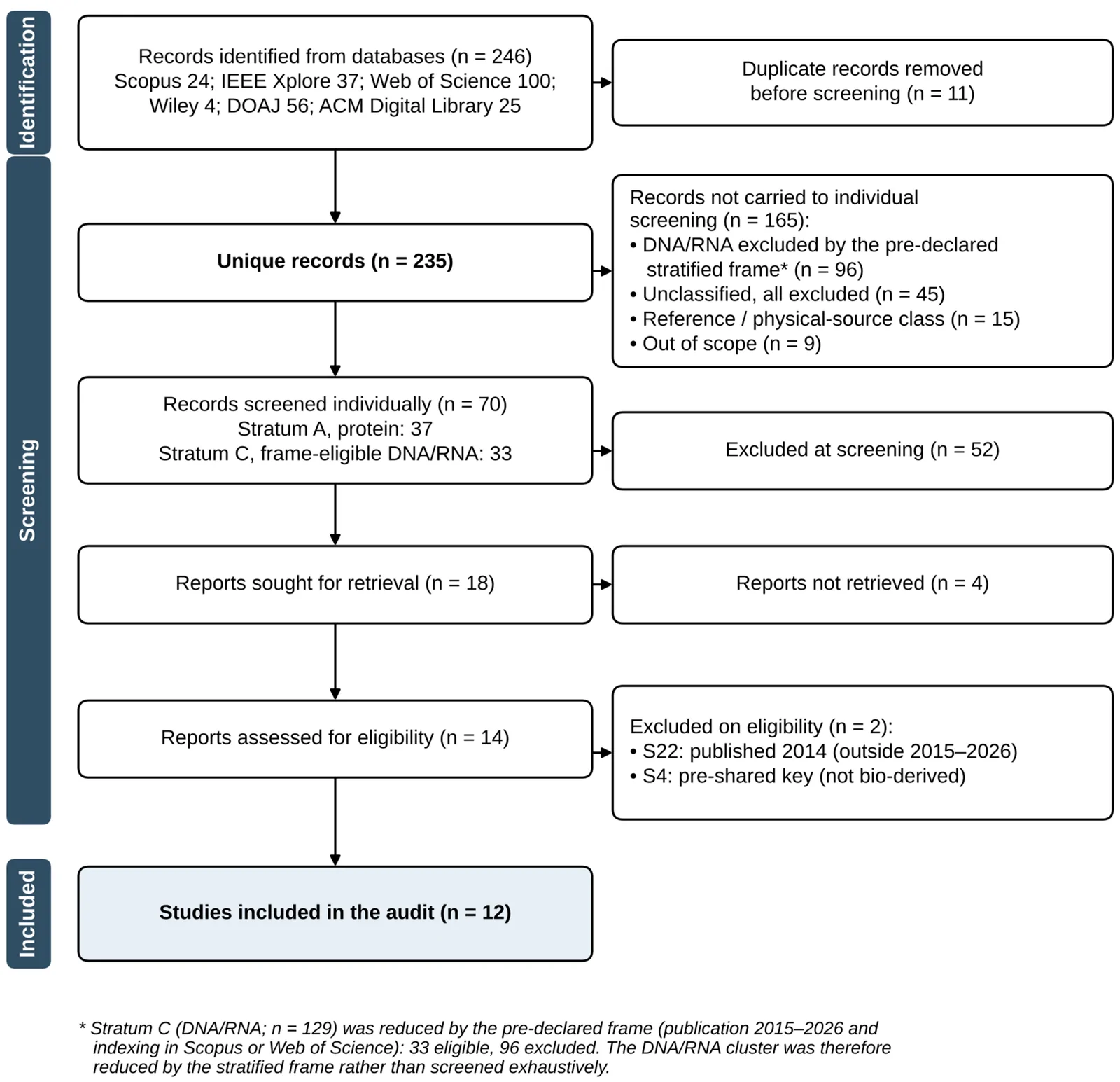
PRISMA 2020 flow diagram for record identification, screening, and inclusion (246 records → 12 included studies). The DNA/RNA (Stratum C) cluster was reduced by the pre-declared stratified frame rather than screened exhaustively.

Records were deduplicated automatically and then by hand. Each record was assigned to its stratum before screening. Title and abstract screening and full-text screening were carried out against the eligibility criteria, with an exclusion reason recorded for each record excluded at individual screening; records removed by the pre-declared Stratum-C frame or as off-topic are marked with the rule applied rather than adjudicated individually. Each included work was then coded on six axes: (1) the biological source and its role; (2) the source category on the five-category scale of Section 4.1; (3) the actual secret, its guessing space in bits, and whether that space is stated by the authors, inferred by us, or absent; (4) the cryptographic structure (key-derivation function, extractor, salt, or secret seed); (5) the attacker model, the security claims, and the evidence offered; and (6) reproducibility: code and data availability and whether a means of verifying a recovered key exists. The 246 records retrieved (Scopus 24, IEEE Xplore 37, Web of Science 100, Wiley 4, DOAJ 56, ACM 25) reduced to 235 unique after removing 11 duplicates. By preliminary stratum these 235 were: protein (Stratum A) 37; DNA/RNA (Stratum C) 129; a biometric/physical reference class 15; out-of-scope 9; and unclassified 45 (37 + 129 + 15 + 9 + 45 = 235). Stratum C was reduced by the pre-declared frame (publication in 2015–2026 and indexing in Scopus or Web of Science) to 33 eligible, with 96 excluded by the frame (published before 2015 or not indexed; 33 + 96 = 129). The 70 stratum-eligible records (37 protein + 33 DNA/RNA) were screened at title/abstract and full text; the 45 unclassified records were screened and all excluded as off-topic, and the 15 reference-class and 9 out-of-scope records were not carried forward. Of the 70 stratum-eligible records screened, 18 proceeded to full-text inclusion and 52 were excluded at screening; of the 18, 4 could not be retrieved in full text (titles logged) and 14 were extracted; two were then excluded on eligibility grounds (S22, published 2014, outside the Stratum-C window; S4, whose key is pre-shared rather than derived from the biological source), leaving 12 included studies. A per-record table accompanies the paper, listing the preliminary stratum of all 235 unique records together with the recorded eligibility decisions and exclusion reasons for the records taken to individual screening; records removed by the pre-declared frame are marked as such. The search and screening followed PRISMA 2020 reporting principles [9]. Because the DNA cluster was reduced by the stratified frame rather than screened exhaustively, this is a structured evidence audit, not a complete systematic review. The complete record flow is shown in Figure 1.

### 3.6. Per-Claim Coding and Agreement

Each security claim is coded separately as Supported, Partially supported, Unsupported, Not applicable, or Insufficiently specified for assessment, with weak security kept distinct from incomplete description; a claim that cannot be judged is not the same as a claim that fails. When a work makes more than one claim, the strongest is the one the authors foreground; where two compete, both are coded. Coding was carried out by a single expert coder against the frozen codebook; a second-pass re-code by the same coder was used to test coding stability, and agreement is reported per axis as test–retest consistency, not as independent inter-rater reliability. A pilot of thirteen papers fixed the codebook; same-reviewer per-axis consistency was measured on the thirteen pilot records (paired units: 13 for eligibility, stratum, and source category; 10 for the strongest-claim outcome): observed agreement 100% on eligibility, stratum, and category (κ = 1.00) and 90% on the strongest-claim outcome (κ = 0.83), above the pre-set transition threshold of 0.60. These are same-coder test–retest figures, not independent inter-rater reliability, and the coding-consistency summary (per-axis agreement) accompanies the paper. A record-flow summary with per-stratum counts and a deviations log accompany the results.

These figures measure the stability of the coding, not independent agreement. The included studies were then screened at full text and coded on the six axes by the same coder against the frozen codebook; the claim-level coding table and the deviations log accompany the paper. Independent multi-coder replication was not performed and is noted as a limitation in Section 8.

## 4. Taxonomy and Security Observations

### 4.1. A Taxonomy of Biological Sources by Role (C1)

A biological input can play one of five roles in a key scheme. The role, not the biology, fixes what the input can guarantee.

- Category 1—public descriptor: The object or its descriptor is taken from a public source available to the attacker—for example a protein structure retrieved from a public archive. It can format or seed a derivation but provides no secrecy, because the attacker holds it.
- Category 2—secret selection from a public database: The secret is the choice of one object from a public database. The uncertainty is the size of the selection set, log2 N bits, not the size of the object. This is the only category in which a public biological object contributes secrecy, and only through the selection.
- Category 3—private sequence or sample: The source is a sample the attacker does not hold, such as an individual’s unpublished sequence. This can carry real secrecy, bounded by the attacker’s uncertainty about the sample and subject to how it is stored and used.
- Category 4—noisy physical measurement: The source is a physical measurement with noise, as in a proteinoid signal or a physical unclonable function. Secrecy is possible but requires entropy accounting and error handling of the fuzzy-extractor kind; this category is read here as a reference class.
- Category 5—metaphor or encoding with no entropy source: The biology is a naming or encoding device (codon tables, a central-dogma analogy, an amino-acid alphabet) with no entropy source of its own. Any secrecy in such a scheme comes from a separate component, commonly a chaotic map or an externally supplied key.

**Figure 2:**
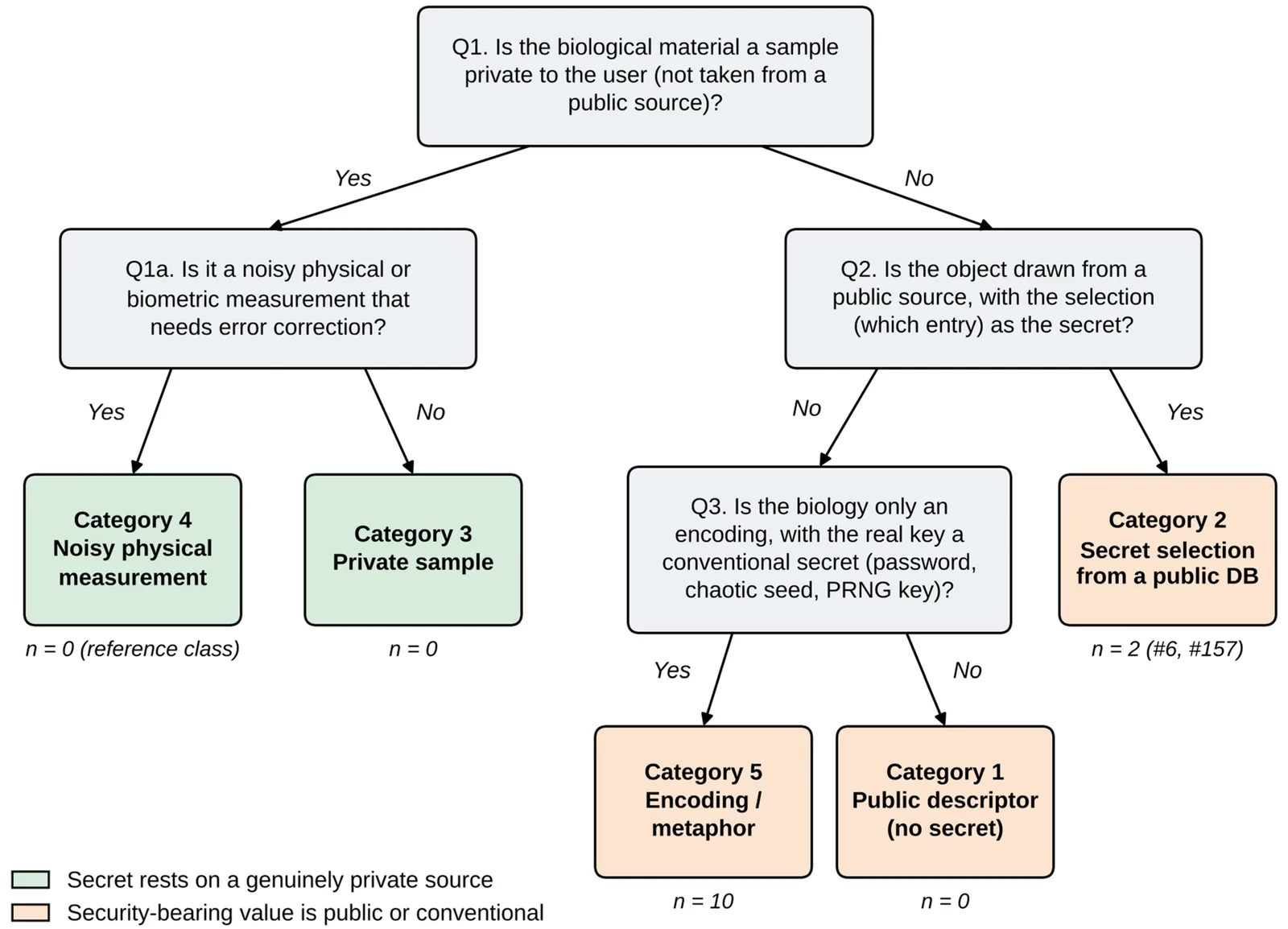
Decision tree for the five-category taxonomy. A scheme is placed by where its security-bearing secret resides; categories 1, 2 and 5 keep that value public or conventional, while categories 3 and 4 rest on a genuinely private source. Shading marks the two groups, and the number of audited studies (n = 12) in each category is given below its box.

The categories are mutually exclusive at the level of the security-bearing component: a scheme is placed by where its secrecy actually resides, not by the biological vocabulary it uses. Where a scheme’s biological object plays one role but its secret resides elsewhere (as in a public descriptor whose only secret is the selection), we record the biological role and the security-bearing secret source as separate fields (Table 1) and place the scheme by the latter. Figure 2 gives a decision tree that applies this classification.

### 4.2. Three Security Observations (C2)

The following statements bound what a biological input can contribute. They are elementary and known to cryptographers, and are stated here because the audited schemes frequently do not apply them; we list them as observations rather than theorems.

- Observation 1

If a key is a deterministic function of a public input, it carries no secrecy. Formally, if K = f(P) with f deterministic and P known to the attacker, then H∞(K | P) = 0: the attacker computes K directly. A key derived by hashing a published structure falls under this observation.

- Observation 2

A public input added to a key-derivation function contributes no secret entropy. If K = KDF(S, P) with P public, then P may act as salt or domain separation, but the secrecy of K is bounded by the conditional min-entropy of S from the attacker’s viewpoint, H∞(S | attacker’s knowledge). Enlarging or complicating P does not raise this bound.

- Observation 3

Descriptor length does not establish key strength. The length of a biological descriptor, or the output length of a hash applied to it, is an upper bound on representation size, not a measure of entropy; deterministic processing cannot introduce entropy absent from the source. This is consistent with the entropy-source guidance [3]: the quantity to report is the min-entropy of the source, not the size of its encoding.

Read together, the three observations identify the single question that decides a bio-derived key claim: what does the attacker not know, and how many bits is it? Section 5 and Section 6 apply this question to the audited schemes and to the key-recovery experiments.

These failure modes are not specific to biology. A password-based key derivation with no salt or stretching, a cypher keyed by a public constant, or a home-made generator seeded by a public value fails the same test: what decides security is the attacker-conditioned min-entropy of the secret, not the size of the output or the apparent complexity of the construction. What the biological framing adds is the appearance of complexity—large descriptors and elaborate encodings—over a secret that is public or conventional. The audit therefore measures a general confusion between representation and secrecy, made vivid by the biological setting rather than caused by it, and the checklist of Section 8 applies to any scheme that derives a key from a public or low-entropy source.

## 5. Audit Results (C3)

Twelve studies passed full-text eligibility and were coded on the six axes of Section 3; two further records were excluded on eligibility grounds recorded in the deviations log—one DNA-coding scheme published in 2014 [10], outside the Stratum-C window (2015–2026), and one steganographic scheme [11] whose key is pre-shared rather than derived from the biological source. Results are reported per source category. With twelve studies the counts are descriptive; we list them as proportions of a stated denominator, not as estimates of a wider population. Table 1 summarises the extraction across all twelve studies; the subsections below give the per-axis findings.

**Table 1:** Summary of findings for the twelve audited studies: source category, the security claim with its reported key space, the attacker-conditioned assessment established in this audit, and the per-study outcome across the 24 coded claims. The full six-axis extraction is provided in the Supplementary Material (Table S1).

Study [Ref]	Cat.	Reported Security Claim/Key Space	Attacker-Conditioned Assessment (This Audit)	Outcome (of 2 Claims)
S2 [12]	5	Robustness to statistical/differential & chosen-plaintext attacks; key sensitivity (NPCR/UACI)	No key-space or min-entropy stated; metrics measure output diffusion, not secret entropy	Partially (2)
S3 [13]	5	Security from NIST randomness (13/14) and ~75% key sensitivity; key is a user password	Output randomness is not key secrecy; no entropy accounting	Unsupported (1), Partially (1)
S5 [14]	5	Key space 2^448^ (~2.3 × 10^121^ yr); entropy ~8; NSCR ≥ 99.57%	~71% of the key is derived mid-encryption from data/encoding, not an independent secret; figure is a codomain count	Unsupported (1), Partially (1)
S6 [15]	2	Key > 6,924,493 bits	Secret = selection among 647 public AlphaFold structures; recovered 647/647; work factor log2(647) = 9.34 bits	Unsupported (1), Partially (1)
S9 [16]	5	Multi-level protection via three hidden keys + DNA/amino stages; PSNR	Design narrative + PSNR; no entropy accounting or attacker model	Unsupported (2)
S12 [17]	5	Key space~product of dynamic encoding tables (26!*26!*26!*10^60^…); codomain 484 bits	Output entropy ≥ input (DNA) entropy, left unestablished; counts a public-table codomain	Unsupported (2)
S15 [18]	5	Key space ~2^797^ (chaotic initial values + amino); resists differential/brute/statistical/noise	Chaotic seed is a genuine secret, but the figure overcounts (full-precision independence + encoding folded in)	Partially (2)
S21 [19]	5	Survives brute force (64-bit GA key); resists COA/KPA/differential	64-bit key asserted random without entropy accounting; attack claims qualitative	Unsupported (2)
S24 [20]	5	Key space up to 20 × 2^471^; round keys pass SP800-22; strict avalanche	2^471^ folds key-derived encoding permutations into the count; independent secret is the 128-bit master key	Unsupported (1), Partially (1)
S31 [21]	5	Brute force 2^224^ (prob 1/2^224^); NPCR ~99.5%, H ~7.96; KPA/CPA/COA/MITM narrative	224-bit figure counts an image-dependent k-mer/encoding arrangement, not an independent secret; parts inferable	Unsupported (1), Partially (1)
S157 [22]	2	~2 × 10^43^ operations (~143 bits)	Secret = reference + position (+16-bit KEK); executed recovery 100/100 ~12.4 bits; 8-ref search space 23.32 bits; ≤39.32 projected	Unsupported (1), Partially (1)
S199 [23]	5	224-bit key -> 2^224^ brute-force security; CPA inapplicable (symmetric)	Public field-size summation conflated with the secret; CPA argument narrative	Unsupported (2)

### 5.1. Distribution Across the Source Categories (RQ1)

The studies concentrate in one category (Table 2). Ten of twelve place the biological material in an encoding or metaphor role with no entropy source (category 5); the remaining two derive the key from a secret selection from a public database (category 2)—a public protein structure in one case (its descriptor is public, so the only secret is the selection) and a public DNA reference in the other. No study in the set is a pure category 1 (a public descriptor used directly as the entire key, with no selection and no conventional secret) or a category 3 (a genuinely private biological sample): the eligible, retrievable schemes derive keys by encoding public biological data or by selecting from public databases, so none placed its secret in a privately held sample and none left the key wholly public without at least a selection or a separate conventional secret. Category-4 (noisy physical measurement) work exists but was pre-declared as a reference class and excluded from the denominator. This absence is itself a finding: the private-sample and well-formed fuzzy-extractor designs that could carry real secrecy are largely absent from the bio-inspired key-generation literature in scope, and the categories that remain are precisely those in which the biological material does not hold the secret. The physical/biometric works identified during screening were held in the reference class and are not counted here.

The concentration in category 5 is itself a result: in most of these schemes the biological element carries no secret, and any security present comes from a separate component—most often a chaotic map or a conventional key. Section 6.4 and the accompanying category-5 accounting quantify this for the encoding schemes examined; the biological input contributes no secret bits in any of them.

### 5.2. The Actual Secret and Its Guessing Space (RQ2)

In the audited set, no study reported a defensible attacker-conditioned min-entropy for its complete secret; where a figure is reported it measures an output or encoding-table size, a descriptor length, or a parameter-range product, and several studies report no key-space figure at all. Reported figures range from a few hundred bits to more than six million; the recovery work factors in Section 6, where a secret could be enumerated, are between about nine and twelve search-space bits for the executed recoveries, with a projected upper bound of at most about forty bits for the broader (unexecuted) eight-reference analysis—all upper bounds on the secret’s min-entropy, since the selection distribution is not known. Where the secret is a conventional key (for example a 256-bit master key feeding a key schedule), the scheme has a real key space, but the reported figure still overstates it and the biological apparatus adds nothing to it. No study reported the min-entropy of its biological input, and none provided an entropy source analysis of the kind standard in the fuzzy-extractor and entropy-source literature.

### 5.3. Attacker Model and Evidence (RQ3)

Attacker models are mostly implicit. The evidence offered is dominated by image or randomness statistics—histograms, correlation coefficients, NPCR/UACI, NIST STS pass/fail—which speak to diffusion and to the randomness of an output, not to the secrecy of a key. Passing a randomness battery was, in several cases, presented as evidence of cryptographic strength; it establishes that an output looks random, not that an attacker cannot reproduce it. Formal reductions or defined adversaries were absent from the set. At the claim level the unit of analysis is the individual security claim, with the author-foregrounded claim taken as the strongest and competing claims coded separately; among the 24 distinct security claims coded across all 12 included studies (two claims per study—the author-foregrounded claim and the next-strongest), 14 were Unsupported and 10 Partially supported, and none was fully supported, the recurrent reasons being an absent attacker model, insufficient description, absence of entropy accounting, and conflation of a public or encoding space with the secret. The claim-level sheet accompanies the paper. Several audited schemes rest their security on chaotic maps; the basic cryptographic requirements for such chaos-based cryptosystems, including a defined key space and resistance analysis, are set out by Alvarez and Li [24] and are largely unmet in the audited set.

### 5.4. Reproducibility (RQ4)

No study in the set released runnable code through a public link (Table 3). Recorded reproducibility statuses were pseudocode, text-only descriptions, or implementations named without a repository (MATLAB, Python with a library, NET with screenshots). This is the gap the present study’s own reproducibility package is built to avoid, and it is the practical reason the field’s security claims have gone unchecked: without code and a verification target, a reader cannot test a derivation against an attacker.

### 5.5. Do Evidentiary Profiles Differ by Category? (RQ5)

The set is too small and too concentrated to compare profiles across categories quantitatively—ten of twelve fall in category 5. Qualitatively, the two selection-based studies (the public protein structure and the public DNA reference) share the same evidentiary shape as the category-5 group: an inflated key-space figure, image or randomness statistics as evidence, and no runnable code. The distinguishing feature of a scheme is therefore not its source category but whether it accounts for the entropy of its secret, which none in the set does.

### 5.6. Summary

Across the twelve studies the pattern is consistent: the biological material rarely holds the secret, the reported key space measures an output rather than an attacker’s uncertainty, the evidence concerns randomness of outputs rather than secrecy, and no study provided a publicly linked runnable implementation. Section 6 turns two of these cases into measured recoveries and bounds the entropy of a third.

## 6. Reproducible Recovery and Entropy-Accounting Experiments (C4)

This section turns the taxonomy of Section 4 into measured results. For three published schemes—the two whose secret is a selection from a public set and one encoding scheme—we re-implement the key derivation as described and state the secret and the attacker under an explicit model, recovering the key by enumeration for the first two and bounding its entropy for the encoding scheme. All results below hold under the stated known-plaintext model and the implementation assumptions recorded in the reproducibility package; the code, candidate lists with snapshot dates and hashes, random seeds, and environment versions are released with the paper, and each re-implementation is checked against a worked example from the original article before it is attacked. The three schemes were chosen by a stated criterion: the security-bearing secret is public, a selection from a public set, or an encoding whose codomain is computable, so an attack is well defined without any private data; the paper specifies the derivation closely enough to re-implement it deterministically; together they cover the categories present—both category-2 schemes (the only ones whose secret is a public selection) and one category-5 encoding scheme for the entropy argument. The remaining nine were not attacked because their security rests on a conventional secret, such as a chaotic seed or a password, that this audit does not claim to break; because they are not specified closely enough to re-implement; or because a recovery would add nothing to the category-level finding already recorded at claim level.

### 6.1. Threat Model and Quantity Measured

The attacker knows the algorithm and every public input: the design, the published encoding tables, and any biological object or database drawn from a public source. The attacker holds a verification target (a ciphertext with a known-plaintext prefix) and tests a candidate against it. A recovery is counted as successful when a candidate reproduces a 16-byte (128-bit) known-plaintext prefix; under an ideal-random, approximately independent wrong-candidate benchmark a wrong candidate passes this test with probability about 2^−128^ (not a guarantee for these schemes, whose candidates can be correlated), and structural collisions (proteins sharing backbone atoms in Scheme #6, for instance) can let an equivalent decrypting key match earlier, so what is recovered is an equivalent key over the tested region, and full-message decryption or a second known-plaintext pair distinguishes the exact secret. Because the recovery returns the scheme’s derived key or keystream, that key decrypts the whole message, not only the matched prefix; the prefix is the test that selects the key, not a limit on what the recovered key covers. The recovery cost is dominated by candidate key-derivation and the known-plaintext check; no multi-threading or optimisation was used, so the reported times are single-threaded upper bounds, and against a naive search over the claimed key length the selection attack is smaller by many orders of magnitude. The quantity of interest is the min-entropy of the secret from the attacker’s viewpoint,

H∞(S) = −log_2_ max_s_ Pr[S = s],

so the best single guess succeeds with probability 2^−H∞(S)^. When the secret is a uniform choice of one item from a candidate set of size N, H∞(S) = log_2_ N and enumeration recovers it after (N+1)/2 guesses on average and N in the worst case. The security ceiling is this selection entropy, which depends on the candidate set the attacker must consider; we therefore report it as a function of that set’s size, not as a single number.

### 6.2. Scheme #6—Key from a Selected Public Protein Structure (Category 2)

Al-Husainy, Al-Sewadi and Sayed [15] build the key from a protein file selected from a public database, mapping each residue to a published code (its published residue-code table) and repeating the table across the image. Their reported key length is

K_bits = ((8 + 8) + (log W + log H + log P)/log 2) × n, n = W·H·P,

giving the reported figure of more than 6.9 × 10^6^ bits for a 236 × 157 × 3 image, with a claimed key space >2^K_bits^. The source is not internally consistent here: evaluating the printed expression at W = 236, H = 157, P = 3 yields about 3.64 × 10^6^ bits rather than the stated 6.9 × 10^6^. Either way the quantity is a representation length obtained by repeating a fixed per-pixel code over the image, not a measure of secret uncertainty. The residue-to-code map is public, so under the stated model the value the attacker must search over is which protein was selected; the ceiling is log_2_ N for the candidate set of size N. The scheme’s amino-acid codes, atom codes, and row permutation are all produced by a pseudorandom generator seeded by the image size n = W·H·P, which is public; our re-implementation uses the paper’s published Table 1 and Table 2 verbatim and reproduces the substitution-and-transposition pipeline as one deterministic instantiation consistent with the published description. Because the paper does not name the pseudorandom generator or provide a complete numeric vector, exact byte-for-byte equivalence cannot be established; however, the published code tables are reproduced verbatim and every derived transform is a deterministic function of the public image size n, so the recovered work factor log_2_ N is invariant to that under-specification. Naming the generator would fix the exact output bytes but not change this count: the generator is seeded only by the public image size, so whichever algorithm it is, it adds no secret. Because every one of these transforms is a deterministic function of the public n, none adds secret entropy.

We verified recovery on a candidate set of N = 647 AlphaFold structures, a documented sample regenerated by the Scheme-#6 notebook, whose accession list, download date, and per-file SHA-256 digests are recorded in the archived reproducibility deposit; under a complete deterministic reconstruction consistent with the published pipeline (both code tables and the transposition), an equivalent decrypting key was recovered in every trial (647/647 = 100%); the work factor is log_2_ N = 9.34 bits for this set. Distinct structures whose residue-code sequences coincide over the bytes used would produce the same keystream, but such collisions only reduce the number of distinct candidates, so log_2_ N is an upper bound on the attacker’s work, not an underestimate. The specific set is not load-bearing: because the work factor is log_2_ N, we report it as a sensitivity over the size of the candidate set the attacker adopts (Table 4): The uniform-selection benchmark is (N + 1)/2 ≈ 324 guesses; the measured mean was lower (91) because proteins sharing their opening backbone atoms yield the same keystream prefix, so a decrypting key is often found before the exact protein, a collision that only shrinks the effective secret space.

The specific value 9.34 bits is the point for the evaluated sample; it is not claimed as the scheme’s general security. The conclusion, however, is stable across the whole range: even if the attacker must search the entire AlphaFold database, the work factor is about 27.7 bits—against a claimed 6.9 million. The overstatement is therefore robust to the choice of candidate set; only its exact size, not its order of magnitude relative to the claim, is open.

### 6.3. Scheme #157—OTP from a Selected Public DNA Reference (Category 2)

Djaa, Mekkaoui and Boukli-Hacene [22] extract one-time-pad subkeys from a DNA reference D selected from a public database. A long integer S is exchanged by Diffie–Hellman; from S the scheme derives a start position POS = S mod length(D) and a 16-bit value KEK (Algorithm 1 of [22]). For each 96-bit plaintext block it extracts m = 80 consecutive bases from D beginning at POS (the first 48 key the plaintext block and the remaining 32 key the generated subkeys), and KEK is applied alongside the DNA-derived keys. The paper reports an attacker effort of ~6 × 10^30^ to 2 × 10^43^ operations, about 143 bits at its top figure. Under Kerckhoffs’s model the reference is public and the secret is the material that fixes the keystream: the start position, the 16-bit KEK, and the run of bases consumed from POS. POS and KEK are both functions of S, but POS = S mod length(D) does not fix the sixteen bits KEK takes from S, so an attacker who fixes the reference and position still faces KEK as a separate unknown of at most sixteen bits. The recovery target is therefore an equivalent decrypting key, the (position, KEK) that reproduces the keystream, tested against a known-plaintext prefix. For a candidate reference set the position search space is

W = Σ_{D∈𝒟} max(0, L_D − m + 1),

with m = 80 the number of bases consumed from POS for a single 96-bit block, which sums the admissible start positions over the references without assuming a common length; the analysis uses the single-block extraction (m = 80) as specified, and does not assume the start position advances across blocks. The executed single-reference recovery used NC_001422.1 (bacteriophage φX174, 5386 bases) and recovered every secret (100/100), fixing the position component at log_2_(L − m + 1) ≈ 12.4 bits. Over a documented snapshot of eight public reference sequences (lengths 5386 to 5,498,578 bases; download date and per-reference SHA-256 digests in the reproducibility package) the admissible-position sum is W = 10,486,338, so log_2_ W = 23.32 bits—a search-space size for the snapshot, not an exhaustively executed recovery. The 16-bit KEK, which the reference and position do not determine, enters the search as an additional unknown of at most 16 bits, so the effective space is at most W·2^16^ and the work factor is

H_search = log_2_(W·2^16^) = log_2_ W + 16 ≈ 39.32 bits

(at most, for the evaluated snapshot).

This is an upper bound on the log-cardinality of candidate (reference, position, KEK) equivalent keys for the evaluated snapshot (a search-space size of 23.32 bits plus the scheme’s own 16-bit KEK), against the paper’s ~143-bit figure. Diffie–Hellman secures the exchange of S but does not enter this count: the attacker does not solve the discrete-logarithm problem, it enumerates the small key material that S expands into and tests each candidate against the known-plaintext prefix. The claimed pool of about 163 million sequences would raise the reference term, but the reported 143 bits counts the combinatorics of the public DNA-to-protein encoding, not the secret; the bound grows only as log_2_ W + 16 and stays far below it. The only executed recovery is the single-reference run above (100/100, ≈12.4 bits); the 23.32-bit and 39.32-bit figures are search-space sizes for the eight-reference snapshot: the product W·2^16^ (about 6.9 × 10^11^ candidates) was not enumerated, and because POS and KEK both derive from S they need not be independent, so W·2^16^ bounds the reachable pairs rather than measuring them. S157 is placed in category 2 by its biological role (a public reference whose selection is the visible secret), while the exchanged integer S is a conventional secret the attack does not recover; what is recovered is the smaller equivalent (reference, position, KEK) key.

### 6.4. Scheme #12—Entropy Upper-Bound Analysis for an Encoding Scheme (Category 5)

Guru Prakash et al. [17] build dynamic character-encoding tables from two DNA sequences and an intron sequence supplied by the sender (Algorithm “Encoding Table (DS_1, DS_2, Collate_Chars)”), and report a key space

K = 26!·26!·10!^6^·26!·26!, log_2_ K ≈ 484 bits.

This is an entropy upper-bound analysis, not a key recovery. K is the number of possible character-encoding tables (the size of the table codomain), not the entropy of the secret that selects one of them. The table is a deterministic function of the inputs (DS_1, DS_2, intron), so the number of tables reachable from a secret of B bits is at most 2^B^; drawing every secret of B bits and counting distinct tables confirmed exactly 2^B^ for B = 8, 12, 16. The effective entropy is therefore the entropy of the DNA inputs, which the paper does not account for: it specifies no source, length, or distribution for DS_1 and DS_2, and gives them no min-entropy analysis. Consistent with Observation 3 (Section 4.2), the reported 484 bits bounds the codomain, not the secret; the scheme’s actual key strength is left unestablished by its own description. We do not attach a single number to it, because the paper provides none to reproduce. The B = 8, 12, 16 check is a sanity test that no collisions arise in a small range, not a measurement of the scheme’s entropy; the substantive result is analytical: the entropy of the encoding output cannot exceed the entropy of the secret DNA inputs, which the paper does not establish.

### 6.5. Across the Three Schemes

**Figure 3:**
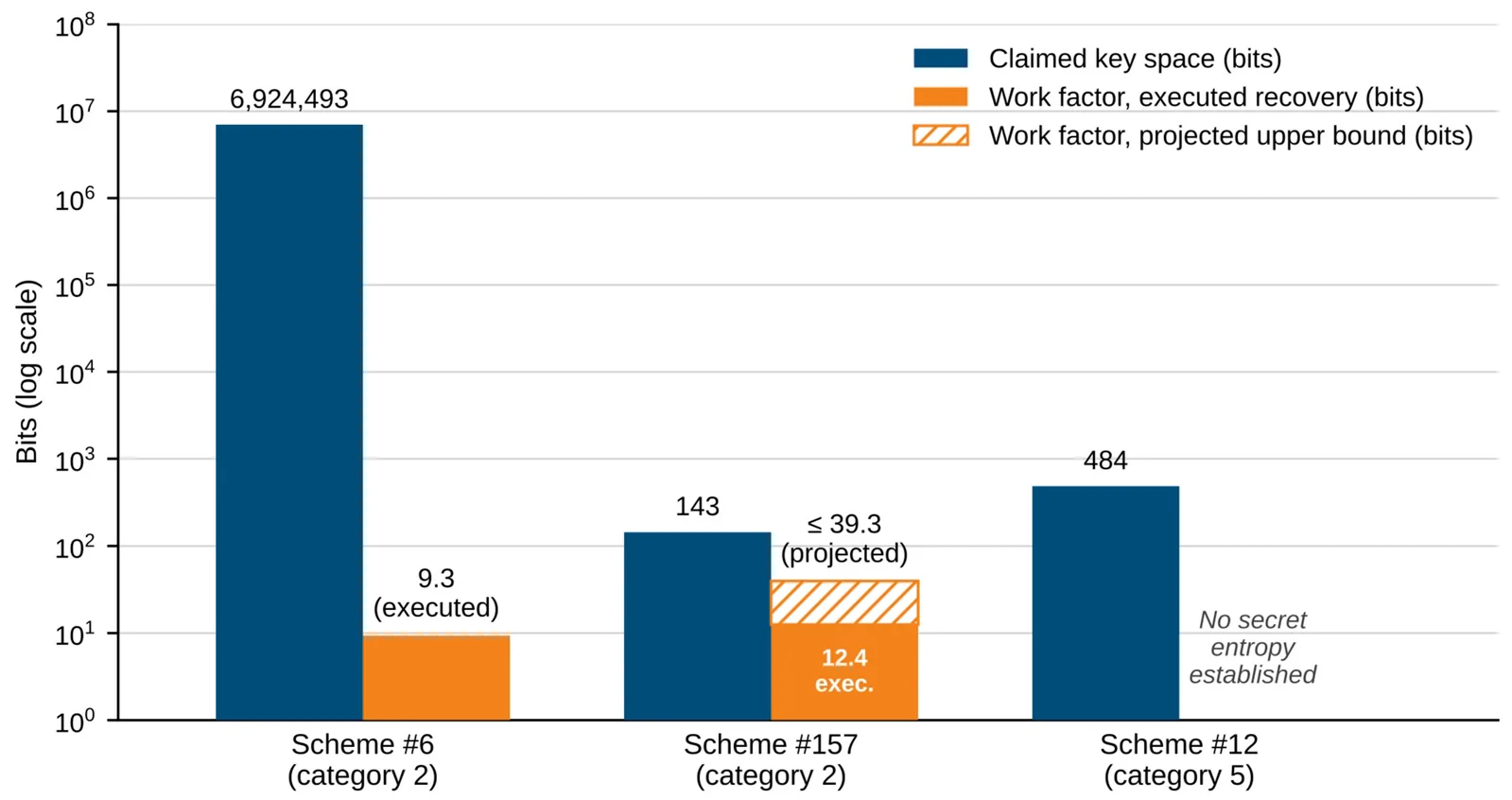
Reported key space versus the work factor established in this study for the three schemes examined (two in category 2, one in category 5; log scale). For #6 the bar is the recovered secret’s search-space work factor (9.3 bits), executed over 647 candidates. For #157 the solid bar is the executed single-reference recovery (≈12.4 bits) and the hatched segment extends it to the projected upper bound (≤39.3 bits = the 23.3-bit eight-reference search-space size plus the 16-bit KEK), which was not exhaustively executed. For #12 the figure is the encoding-table codomain (484 bits); the paper specifies no source or distribution for its DNA inputs, so no secret entropy is measured and no work-factor bar is shown.

The same relation holds under the stated model in every case (per-run metrics in Table 5; summarized in Table 6 and Figure 3): the reported key space equals the size of the output, or of the space of all possible tables, while security is bounded by the reachable secret. For the deterministic derivations considered here, where the key is a function K = f(S, P) of a secret S and public inputs P, public deterministic processing cannot increase the attacker-conditioned uncertainty of S, so the reachable key space is bounded by the secret space:

H∞(K | attacker) ≤ H∞(S | attacker) ≤ log_2_ | reachable secret space |.

The gap between the reported figure and the reachable secret is the public component that each scheme counts as if it were secret. The recovery attacks apply where the secret is a public object or a selection from one. They do not apply to a scheme whose secret is a private sample unavailable to the attacker (category 3), which is the boundary of this study, nor do the numerical results extend beyond the known-plaintext model and the implementation assumptions documented with the code.

## 7. Lessons from Biometrics and Physical Entropy Sources

Turning a noisy or partly public biological signal into a key has an established treatment in a neighbouring field, and that treatment separates the quantities the audited schemes run together. A fuzzy extractor maps a noisy source W to a near-uniform key R together with public helper data P, and its analysis keeps four objects distinct—the source, its min-entropy, the public helper P, and the key R—accounting for the leakage through P rather than assuming it is zero [1,2]. Physical unclonable functions and physical randomness extractors share the same shape: security is tied to a measured, non-reproducible physical quantity and an estimate of its entropy, not to the size or intricacy of a descriptor. Proteinoid and wet-lab bio-cryptography resemble this physical setting more than they resemble key derivation from a public sequence, which is why such work was held in the reference class (Section 3).

This discipline—source, min-entropy, public helper, near-uniform key—is exactly what the audited schemes omit: where a biometric construction reports the min-entropy of its source and the leakage of its helper data, the schemes of Section 5 report a descriptor length, a hash-output length, or a permutation-space size and call it the key space. The lesson is standard and available; citing the correct source for each claim—the fuzzy-extractor line for noisy sources, the entropy-source guidance for deterministic post-processing—is enough to locate where a scheme’s accounting breaks. The design checklist in Section 8 turns this into a short list of checks a scheme author can apply before submission.

## 8. Design Checklist, Limitations, and Conclusions

### 8.1. A Design Checklist (C5)

The checklist below turns the observations of Section 4 and the results of Section 5 and Section 6 into questions an author can answer about their own scheme before submission. It is specific to bio-derived key schemes, not generic security advice; a scheme that cannot answer the first three questions in bits has not established a key space.

1. State the secret. What exactly does an attacker not know—a sequence, a selection, a seed, a measurement? Name it before anything else.
2. Locate it relative to public data. Is the biological object, or the choice of it, drawn from a public database? If so, the secret is the selection, and its size is log2 N for a set of N candidates—not the size of the object.
3. Give the min-entropy in bits. Report H∞(S) from the attacker’s viewpoint. If it cannot be stated, the key space is unestablished, whatever figure the construction suggests.
4. Do not confuse length with entropy. Descriptor length, hash-output length, or the number of possible tables is an upper bound on representation, not a measure of secret uncertainty.
5. Provide a verification target and test recovery. If a ciphertext, tag, or transcript lets a key be checked, enumerate the secret and report whether it can be recovered, with the guess count.
6. Separate secrecy evidence from randomness evidence. Histograms, correlation, NPCR/UACI, and NIST batteries show that an output looks random; they do not show that an attacker cannot reproduce the key.
7. Release code and a data snapshot. Publish the derivation, the candidate list with dates and hashes, and one runnable example, so the claim can be checked rather than taken on description.

### 8.2. Limitations

The audited set is small (twelve studies) and concentrated in one category; the counts in Section 5 are descriptive, not estimates of a wider population. No scheme in the set falls in category 3 (a private sample) or category 4 (a noisy physical measurement); category-4 work was held in the reference class (Section 3). The findings therefore bound schemes whose secret is public, a selection from public data, or an encoding, and do not speak to a well-designed construction that draws its secret from a genuinely private sample or that treats a physical source with the fuzzy-extractor discipline of Section 7—such a scheme can carry real secrecy and lies outside this study. The recovery experiments hold under a known-plaintext model and the implementation assumptions in the reproducibility package, and they apply where the secret is a public object or a selection from one; they do not extend to a private-sample scheme (category 3). The category-5 result is an entropy upper-bound, not a full recovery. The reported work factors are for the evaluated snapshots, with sources and dates recorded. Coding was performed by a single expert coder with a documented test–retest consistency check; independent multi-coder replication was not performed and is a limitation. The claim-level proportions should therefore be read as the assessment of one expert reader rather than as agreement established across independent coders, and the field-level conclusions are qualified accordingly. Four eligible records could not be retrieved in full text and were excluded; with a small sample this may introduce an availability bias, and their titles are logged so the omission is transparent. Claim-level coding was completed for all twelve included studies (24 claims); the proportions are reported descriptively and are not generalised beyond the audited set. This is a structured evidence audit rather than a complete systematic review: the DNA-cryptography cluster was reduced by a pre-declared stratified frame, the record flow is reported following PRISMA 2020 principles, and coding was by a single coder. The audit’s role is to structure the evidence and motivate the reproducible key-recovery experiments (Section 6), which are the paper’s primary empirical contribution. The database queries as executed carried an operator-precedence ambiguity in two sources (Web of Science and DOAJ), where the security constraint groups with one term branch rather than with both; the search is reported as executed, with the deviation logged, and a corrected-grouping sensitivity search was not performed. For the ACM export the server-side AND did not apply and crypto-relevant records were retained programmatically; this is logged as a deviation.

### 8.3. Conclusions

Biological complexity does not by itself imply cryptographic secrecy. Across the audited schemes, no reported key-space figure was a defensible attacker-conditioned min-entropy of the secret; each measured an output or encoding-table size, a descriptor length, or a parameter-range product; the biological material most often carries no secret at all, and where a real key exists it is a conventional quantity reachable without the biology. The two key recoveries and one entropy upper-bound make this concrete: executed recovery work factors of about nine to twelve search-space bits and a projected upper bound of at most about forty bits, and an unestablished input entropy, against reported figures of hundreds to millions of bits. The framework that separates output size, encoding codomain, and attacker-view secret entropy, together with the checklist, lets an author, a referee, or a reader check a bio-derived key scheme against the one question that decides it: what does the attacker not know, and how many bits is it. For the biomimetic design community, the lesson is that imitating the complexity of a biological system is not the same as inheriting its unpredictability: a bio-inspired key is secure only when the biological element supplies secret, attacker-unknown entropy, not merely structure.

## Figures and Tables

**Table 2:** Distribution of the twelve included studies across the source categories.

Category	Count (of 12)
1—public descriptor	0
2—secret selection from a public database	2
3—private sequence or sample	0
5—encoding or metaphor, no entropy source	10

**Table 3:** Reproducibility status of the twelve included studies.

Reproducibility Status	Count (of 12)
Public runnable code (with link)	0
Pseudocode	4
Text-only description	2
Named implementation, no public link (MATLAB/Python/.NET)	4
Insufficient to reproduce	2

**Table 4:** Work-factor sensitivity for scheme #6 as a function of the attacker’s candidate-set size N.

Candidate Set N	Work Factor log_2_N (Bits)	Mean Guesses (N + 1)/2
647 (evaluated sample)	9.34	324
10^3^	9.97	500
10^4^	13.29	5000
10^5^	16.61	50,000
10^6^	19.93	500,000
10^8^	26.58	50,000,000
≈2.14 × 10^8^ (full AlphaFold DB [25])	27.67	1.07 × 10^8^

**Table 5:** Recovery metrics for the C4 experiments.

Scheme	Cat.	Secrets Tested	Success	Guesses (Mean/ Median/Max)	Time Per Recovery	Peak Memory	Work Factor (Bits)
#6	2	647	100%	91/27/628	59.9 ms	295 KiB	9.34 (27.67 for the full AlphaFold DB)
#157	2	100	100%	3042/3250/5231	247.8 ms	190.4 KiB	12.4 executed (1 reference); 23.32 search space and ≤39.32 projected (8 references + 16-bit KEK)
#12	5	—	—	—	—	—	≤484 (codomain); input entropy unestablished

Environment: Google Colab, Python 3.13.15, Linux x86_64, 2 vCPU, single-threaded. #6: the measured mean guess count (91) is below the uniform benchmark (N + 1)/2 ≈ 324 because proteins sharing opening backbone atoms give colliding keystream prefixes; the log_2_ N = 9.34-bit work-factor ceiling is unaffected. #157: time and guess counts are per recovery on a single reference (NC_001422.1; feasibility), while the projected work factor is over the eight-reference snapshot (W = 10,486,338, log_2_ W = 23.32) plus the 16-bit KEK and was not executed. #12 is an entropy upper bound, not a timed recovery. The primary measure is the candidate count and work factor; the times are exploratory and specific to the stated environment (no warm-up or dispersion statistics), not a general performance estimate.

**Table 6:** Claimed key space versus the work factor established in this study for the three schemes examined (two in category 2, one in category 5).

Scheme	Category	Result	Claimed Key Space	Established in This Work
#6	2 (public selection)	Key recovery	>2^6.9×10^6^^	log_2_ N = 9.3 bits (647-structure sample, executed); ≤27.7 bits (full AlphaFold DB)
#157	2 (public selection)	Key recovery	≈2^143^	12.4 bits (1 reference, NC_001422.1, executed); ≤39.32 bits projected (8-reference snapshot 23.32 + 16-bit KEK, not executed)
#12	5 (encoding)	Entropy upper bound	2^484^ (codomain)	Input entropy not established by the original paper

## Data Availability

The reproducibility package is supplied as Supplementary Material with this submission. It contains the per-scheme notebooks (executed in a clean environment, Python 3.13.15, with outputs saved), a script that regenerates the comparison figure, the candidate manifests (manifest_s6.csv and manifest_s157.csv, each file with its byte length and SHA-256 digest and download date), the deduplicated claim-level coding table, the search and deduplication logs, the per-record screening table, the deviations file, the comparison figure, an environment specification, and a SHA-256 checksum manifest of all packaged files. Public sequences are fetched by accession from AlphaFold/EBI and NCBI at run time, so the reported candidate set (W = 10,486,338 for Scheme #157) and the per-file digests reproduce. Each re-implementation was checked against the published components that were sufficiently specified before it was attacked. For Scheme #6, exact byte-for-byte equivalence could not be established because the original article did not identify the pseudorandom generator or provide a complete test vector; the experiment therefore evaluates one deterministic instantiation consistent with the published description.

## Supplementary Materials

The following supporting information can be downloaded at https://www.mdpi.com/article/10.3390/biomimetics11100741/s1.
